# Substrate-Dependent Assembly of the Tat Translocase as Observed in Live *Escherichia coli* Cells

**DOI:** 10.1371/journal.pone.0069488

**Published:** 2013-08-02

**Authors:** Patrick Rose, Julia Fröbel, Peter L. Graumann, Matthias Müller

**Affiliations:** 1 Institute of Biochemistry and Molecular Biology, Zentrum für Biochemie und Molekulare Zellforschung, University of Freiburg, Freiburg, Germany; 2 Faculty of Biology, University of Freiburg, Freiburg, Germany; 3 LOEWE Center for Synthetic Microbiology (SYNMIKRO) and Department of Chemistry, University of Marburg, Marburg, Germany; Centre National de la Recherche Scientifique, Aix-Marseille Université, France

## Abstract

The twin-arginine translocation (Tat) pathway guides fully folded proteins across membranes of bacteria, archaea and plant chloroplasts. In *Escherichia coli*, Tat-specific transport is executed in a still largely unknown manner by three functionally diverse membrane proteins, termed TatA, TatB, and TatC. In order to follow the intracellular distribution of the TatABC proteins in live *E. coli* cells, we have individually expressed fluorophore-tagged versions of each Tat protein in addition to a set of chromosomally encoded TatABC proteins. In this way, a Tat translocase could form from the native TatABC proteins and be visualized via the association of a fluorescent Tat variant. A functionally active TatA-green fluorescent protein fusion was found to re-locate from a uniform distribution in the membrane into a few clusters preferentially located at the cell poles. Clustering was absolutely dependent on the co-expression of functional Tat substrates, the proton-motive force, and the cognate TatBC subunits. Likewise, polar cluster formation of a functional TatB-mCherry fusion required TatA and TatC and that of a functional TatC-mCherry fusion a functional Tat substrate. Furthermore we directly demonstrate the co-localization of TatA and TatB in the same fluorescent clusters. Our collective results are consistent with distinct Tat translocation sites dynamically forming *in vivo* in response to newly synthesized Tat substrates.

## Introduction

The Tat machinery is a protein translocation system operating in the plasma membranes of bacteria, archaea and the thylakoidal membranes of plant chloroplasts. The two hallmarks of Tat client proteins are unique signal sequences with a conserved twin-arginine-containing sequence motif and the fact that many of them are translocated only after they underwent complete folding in the cytosol (for most recent reviews see 1–3.

Tat substrates are recognized and translocated by members of the TatA-type and TatC-type families of membrane proteins. TatA-type proteins are composed of an N-terminal transmembrane helix (TDH) that is followed by an amphipathic helix (APH) and an unstructured C-terminal region. Solution [4,5] and solid state NMR [6] of TatA orthologues revealed a TMH being too short to span the bilayer entirely and an angled orientation of the APH that likely leads to contacts of the APH with the membrane lipids. Although recent data strongly re-emphasize an N_out_-orientation of TatA [7,8], the possibility of a complete or partial topology switch of TatA during the transport event has repeatedly been brought up [9,10]. TatC, on the other hand, consists of six TMHs and recent detailed structural analyses revealed an almost complete immersion in the lipid bilayer of the entire molecule including the TMH-connecting loops [11]. Whereas Gram-positive bacteria possess Tat machineries made from one orthologue each of the TatA- and TatC-families, many Gram-negative bacteria and plant chloroplasts express a second TatA-type paralogue usually termed TatB (Hcf106 in plants). Although the *E. coli* TatB shares about 20% sequence identity with TatA [12], it is functionally diverse from TatA [13] and in the absence of TatB, the Tat machinery of *E. coli* can sustain activity only after TatA acquired distinct mutations [14,15]. *Enterobacteria* express in addition an even more homologous paralogue of TatA, termed TatE, which can partially replace TatA [16,17] but is dispensable, when TatA is expressed. The physiological meaning of expressing a *tatE* allele is not understood.

All Tat-family members have the conspicuous propensity to form homo- and hetero-oligomers. TatA is usually purified in detergent as homooligomeric assemblies of varying size (for references see 2), which upon analysis by single particle cryo-electron microscopy show pore-like structures of varying diameters [18]. This finding is the basis of a model, according to which the TMHs of varying numbers of TatA protomers assemble into size-fitting, ring-like pores. Although association of neighboring TatA molecules via their TMHs has been documented by various methods such as site-specific EPR-spin labeling [19] and cross-linking [20–22], it is difficult to imagine how the mostly hydrophobic TMHs would allow a hydrophilic substrate protein to cross the membrane. Nor is the original model consistent with the much smaller pores of isolated TatE complexes that nevertheless can functionally substitute for TatA [17]. An alternative pore-model suggests that rather than the TMHs of TatA, its APHs could fashion a hydrophilic channel. In this scenario, the APHs of TatA would have to flip into the membrane like a “trapdoor” [23]. This view has recently been re-emphasized by the finding that oppositely charged clusters on the APH of TatA and its adjacent C-terminal tail were ideally located to force the formation of hairpins for membrane insertion and to even “zip” up with neighboring TatA protomers to self-assemble into oligomeric arrays [24]. A third model, of how TatA might facilitate the transmembrane movement of Tat substrate proteins, is the “membrane-weakening” hypothesis [25,26] proposing that TatA oligomers could destabilize the bilayer, possibly by membrane-thinning or even membrane-rupturing effects due to the short TMH of TatA [5]. Whilst the molecular details of the actual Tat-specific translocation step have thus remained obscure, it is well established that translocation requires the proton-motive force (PMF).

Whereas TatB and TatC by themselves also show the property of self-association [27–30], when expressed together they form 1:1 complexes [31]. TatBC dimers function as receptor complexes for RR-signal peptides in such a way that TatC exposes a by now well-established, surface-exposed binding site for the N-proximal RR-consensus motif [11,32,33] and together with its associated TatB protomer provides a binding pocket for N-distal sequence regions of RR-signal peptides [34–39]. Recently, we have provided evidence that TatC also functions as an insertase for Tat-signal sequences and that TatB regulates the extent of insertion [40]. Although single TatBC dimers can bind one precursor molecule each [41,42], there is experimental evidence for the existence of heptameric or octameric TatBC complexes with correspondingly more precursor binding sites [41,43]. These oligomeric complexes seem to enable even the simultaneous translocation of several precursor molecules [42] after they associated with multiple TatA protomers. These studies altogether suggest that Tat translocases are huge molecular machineries. Here we demonstrate that such large multimeric Tat translocation sites are in fact formed *in vivo* and for the first time provide evidence that their formation is triggered by the Tat substrates.

## Results

### Tracking Export of a Tat Substrate to the Periplasm of *E. coli* Cells

**Figure 1:**
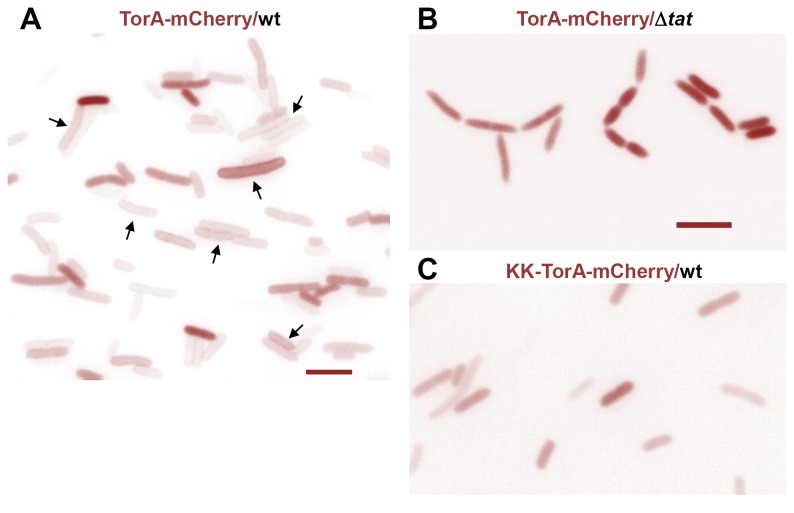
Visualizing transport of the Tat substrate TorA-mCherry into the periplasmic space of *E. coli* cells. (**A**) Fluorescence micrograph of *E. coli* BL21(DE3) cells expressing TorA-mCherry in the presence of the chromosomally encoded TatABC proteins. TorA-mCherry was expressed from a pET22b+-based plasmid (pPJ3) via the basal level of T7 RNA polymerase, which is synthesized in BL21(DE3) cells under non-inducing conditions and yet yields a sufficiently high fluorescence signal. Rim staining by TorA-mCherry (arrows) can be observed in most of the cells. Bar, 5 µm. (**B**) Expression of TorA-mCherry in the *tatABCD* deletion strain BL21(DE3) Δ*tat*. The Tat-deficient cells are typically arranged in chains of non-separated cells and the fluorescent signal of TorA-mCherry is spread throughout the cytoplasm. (**C**) Expression of the non-transportable KK-variant of TorA-mCherry in *tatABC* wild type cells also leads to the retention of the fluorescent substrate in the cytosol.

In order to explore fluorescence microscopy for the visualization of the Tat machinery in live *E. coli* cells, we first inspected *tat* wild-type cells, which had been transformed with a pET-vector encoding TorA-mCherry (pPJ3). TorA-mCherry is a fusion protein between the RR-signal sequence of the natural *E. coli* Tat substrate TMAO reductase (TorA) and the fluorescent protein mCherry. This Tat model protein is efficiently recognized and transported by the *E. coli* Tat translocase as previously shown both *in vitro* [21,32,40] and *in vivo* [40]. Figure 1A depicts an epifluorescence image of *E. coli* cells expressing TorA-mCherry through a basal activity of the T7 promoter. The majority of cells show a uniform rim staining (arrows) suggesting that the bulk of TorA-mCherry had accumulated at the cell envelope. By contrast, the TorA-mCherry-dependent fluorescence was uniformly spread over the cell bodies when the bacteria did not express TatABC (Figure 1B). The phenotype of these *tat* deletion mutants manifests itself by a lack of septation [44] leading to chains of cells, in which the “fluorescent gaps” between adjacent cells are consistent with the absence of TorA-mCherry from the cell envelope. A similar uniform distribution of TorA-mCherry in the cytosol was seen in *tat* wild-type cells that, however, expressed a transport-defective KK-variant of TorA-mCherry (Figure 1C). Hence rim-staining required both a functional Tat translocase and Tat signal sequence and therefore most probably reflects Tat-dependent translocation of TorA-mCherry into the periplasmic space of the *E. coli* cells. Mere binding of TorA-mCherry to the cytosolic leaflet of the inner *E. coli* membrane, either via the TatBC receptor complex or membrane lipids [45,46], could be ruled out as the cause for the rim-staining. In a *tatAE* deletion strain expressing only the TatBC receptor subunits, TorA-mCherry did not accumulate at the cell periphery but uniformly stained the cytosol exactly as in any other individual *tat* mutant (Figure S1) and the global Δ*tatABC* strain shown in Figure 1B. These data strongly suggest that rim-staining by TorA-mCherry was due to its export to the periplasm, as demonstrated also by pulse labeling of TorA-mCherry in intact cells [40], and that by this experimental set-up we were able to follow Tat-dependent transport across the inner membrane of live *E. coli* cells.

### TatBC-dependent Clustering of TatA-GFP

**Figure 2:**
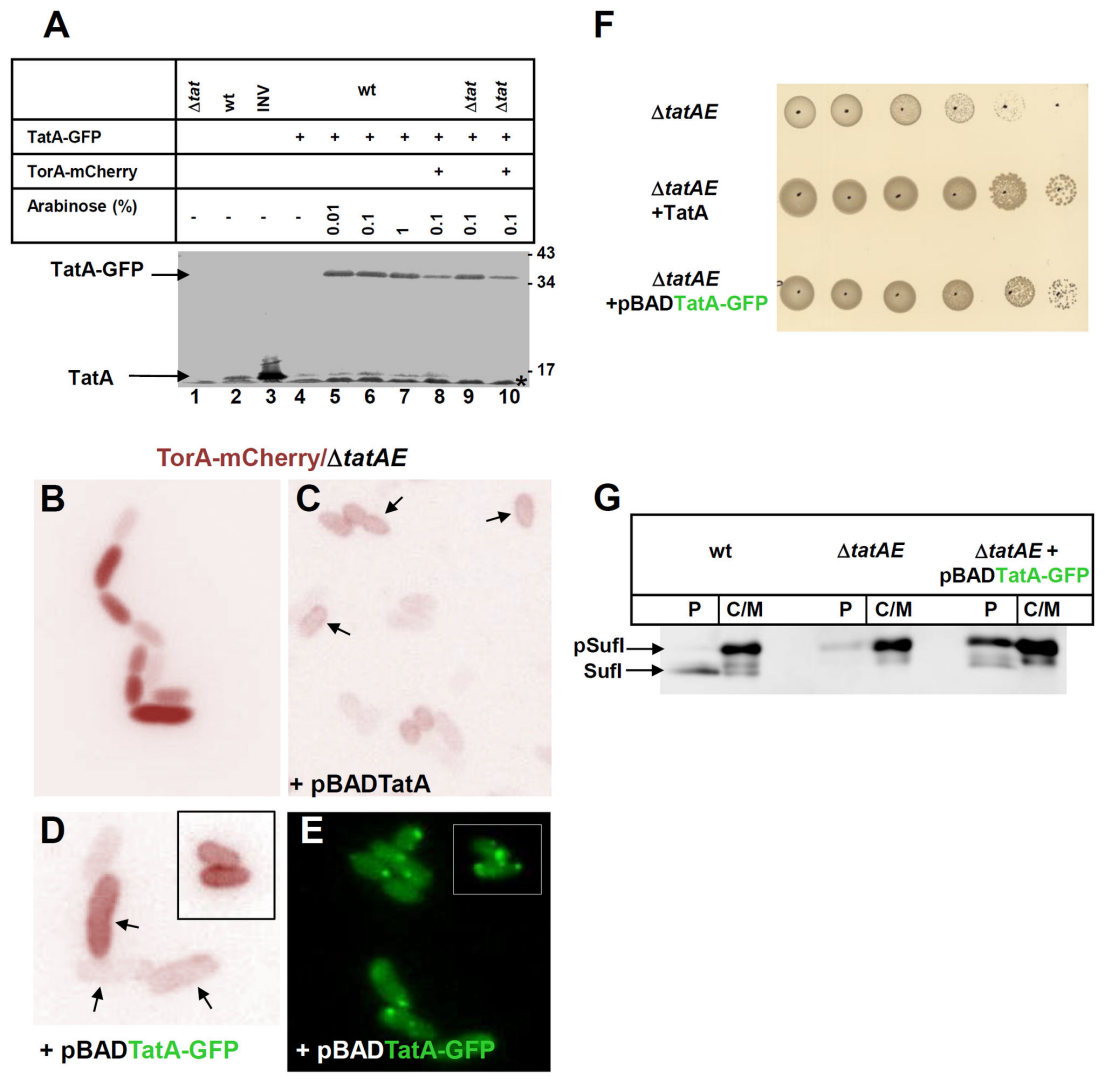
The TatA-GFP fusion is stable and functionally active. (**A**) TatA-GFP was expressed in *E. coli* BL21(DE3) cells from a pBAD33 vector (pPR1) following 2 h of induction by the indicated amounts of arabinose. Whole cell proteins were precipitated with trichloroacetic acid and proteins were separated by SDS-PAGE. Shown is an immunoblot decorated with antibodies against TatA. TatABC wild type cells (wt) show a TatA signal at about 15 kDa (arrow) that is absent from *tatABC* deletion cells (Δ*tat*) and enhanced in inner membrane vesicles (INV), which had been prepared from a TatABC-overexpressing strain. Cells induced with arabinose display a TatA-GFP band at about 36 kDa. Expression of TatA-GFP was slightly reduced by the co-expression of TorA-mCherry from plasmid pPJ3 (lanes 8 and 10). * non-specific band. (**B**–**D**) Expression of TorA-mCherry-SsrA from plasmid pPR8 in JARV16 (Δ*tatAE*) *E. coli* cells and the indicated transformants thereof. In the absence of TatAE, the Tat substrate remains cytosolic and cell separation is impaired (B). Transport of TorA-mCherry to the periplasm (arrows) and cell separation are restored by co-expression of TatA from plasmid pPR4 (C) and of TatA-GFP from plasmid pPR1 (D, inset shows cells from a different picture) demonstrating functionality of the TatA-GFP fusion. (**E**) Cells as in (D) now showing distribution of TatA-GFP. TatA-GFP forms similar clusters as when co-expressed with TatA (cf. Figure 3). (**F**) Growth of cell colonies on LB agar plates containing 2% SDS and 0.1% arabinose. Cells grown in LB liquid media were serially diluted and 5µl were each applied to the agar plate. Both TatA and TatA-GFP complement the growth defect of the Δ*tatAE* mutant strain JARV16 on 2% SDS. (**G**) Immunoblot of subcellular fractions decorated with antibodies against SufI. The strains MC4100 (wt), JARV16 (Δ*tatAE*) and JARV16/pPR1 (Δ*tatAE+*pBAD33TatA-GFP) were transformed with plasmid pPJ9 expressing the native Tat-substrate SufI. Cells were grown at 37° C and 180 rpm to an OD_600_ = 0.3 in LB-medium, supplemented with 0.1% L-arabinose for 1.5 h to induce synthesis of TatA-GFP and subsequently with 50 ng/mL anhydro-tetracycline for an additional 1.5 h to induce synthesis of SufI. Fractionation was carried out as described in Materials and Methods. Periplasmic fractions (P) and cytoplasm/membrane fractions (C/M) were loaded in a ratio 40:1 onto an 8% SDS-gel.

Our major goal was to investigate whether the Tat transport event influences the distribution of the TatABC proteins in *E. coli* cells. To first follow the intracellular localization of TatA, we attached the GFP-moiety to the C-terminus of TatA via a twelve amino acid-long linker. In this way we sought to minimize perturbation of the folding of TatA by the GFP moiety. The *tatA-GFP* fusion gene was cloned into the pBAD33 vector under the control of the *ara* promoter (pPR1). When transformed into a *tatABC* wild-type strain, induction with 0.1% arabinose for 2 h led to a clear over-expression of TatA-GFP over the chromosomally expressed endogenous TatA (Figure 2A, lane 6). Virtually unchanged levels of endogenous TatA before and after induction (Figure 2A, lanes 4 and 6) indicate a high stability of the TatA-GFP fusion protein not being cleaved in the linker region to any considerable extent.

To demonstrate the functionality of our TatA-GFP fusion, we made use of the *tatAE* deletion mutant JARV16, which due to a lack of any functional TatA paralogue does not export plasmid-encoded TorA-mCherry to the periplasm and shows the general *tat* phenotypic chain formation (Figure 2B). Transport was, however, restored to this Δ*tatAE* mutant by complementation with a pBAD vector containing the *tatA* gene (pPR4). Two hours of induction with 0.1% arabinose led to the re-appearance of TorA-mCherry at the cell periphery (Figure 2C, arrows; note also restoration of cell division). Rim-staining by TorA-mCherry was similarly restored by replacing TatA with TatA-GFP (Figure 2D, arrows) indicating that cells, which contained *tatA-GFP* as the only *tatA* allele, in fact sustained Tat-specific export. In addition, *E. coli* cells lacking a functional Tat translocase display impaired growth in the presence of 2% SDS due to alterations in their outer membrane [47]. This phenotype is illustrated in Figure 2F, in which the *tatAE* double deletion mutant shows a growth defect on agar plates containing 2% SDS that is overcome by an extra-chromosomal copy of *tatA*. A similar extent of growth restoration was observed when Δ*tatAE* cells were expressing TatA-GFP instead of TatA. Furthermore we find that TatA-GFP partially restores processing and export to the periplasm of the natural Tat substrate SufI in a *tatA*-defective background (Figure 2G). Thus the TatA-GFP fusion by itself clearly exhibits TatA activity, though to a lesser extent than wild-type TatA.

**Figure 3:**
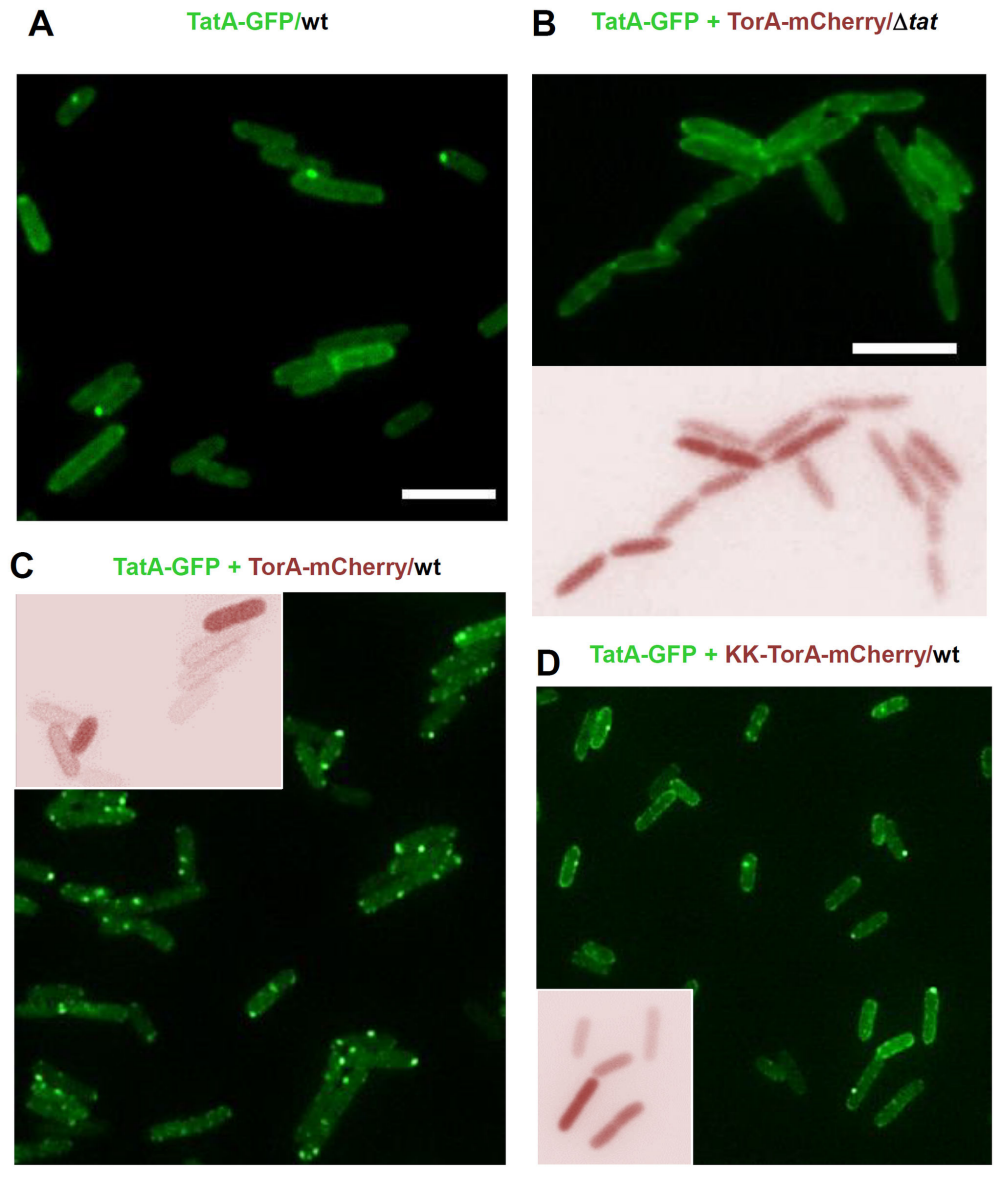
Substrate-dependent clustering of TatA-GFP. (**A**) *E. coli* BL21(DE3) wild type cells (wt) expressing TatA-GFP from plasmid pPR1. Expression was induced for 2h by the addition of 0.1% arabinose. The fluorescence signal of TatA-GFP is distributed evenly in the cell membrane with some tendency to cluster at the cell poles. (**B**) Clustering of TatA-GFP is totally missing in BL21(DE3) Δ*tat* cells lacking a functional Tat-translocase (Δ*tat*). TorA-mCherry co-expressed in the same cells from plasmid pPJ3 is not exported but remains cytosolic (lower panel). (**C**) Co-expression of TatA-GFP and TorA-mCherry in BL21(DE3) wild type cells (wt) harboring a functional Tat-translocase increases the number of TatA-GFP clusters while allowing transport of TorA-mCherry to the periplasm (inset). (**D**) Increased clustering of TatA-GFP in wild type cells is not observed when co-expressed with the non-transportable (cf. inset) KK-variant of TorA-mCherry from plasmid pPJ5.

When cells, which expressed TatA-GFP in addition to the wild-type levels of the native TatABC proteins, were inspected under the microscope, the GFP-fluorescence also accumulated predominantly at the cell peripheries (Figure 3A), similar to what had been described in previous studies using similar TatA-XFP fusions [48,49]. As depicted in Figure 3A, a certain number of cells consistently contained bright single dots, which again is in accordance with previous observations [48,49]. Those prominent dots were virtually absent from cells expressing TatA-GFP in the absence of the chromosomal *tatABC* genes (Figure 3B, upper panel). Rather TatA-GFP was enriched at the cell periphery in a halo-like manner when the TatBC subunits were missing. This distribution pattern was independent of the co-expression of a Tat substrate (compare Figure 3B, upper panel, with Figure S2A). The lack of a functional Tat machinery in these Δ*tat* cells is indicated by their chain-forming phenotype as well as by the defective export of the co-expressed TorA-mCherry leading to its retention in the cytosol (Figure 3B, lower panel). A similar dependence of dot formation by TatA-GFP on the presence of other Tat subunits was previously reported for *B. subtilis* TatA_d_-GFP and the cognate TatC_d_ [50]. Moreover, similar bright foci were observed in *E. coli* cells expressing a TatA-YFP fusion only from the native *tatA* promoter [51]. Hence the dot formation depicted here in Figure 3A could well be explained by a clustering of TatA-GFP at TatBC complexes as a consequence of the assembly of functional TatABC translocases. Those translocase complexes would manifest the ongoing transport of the endogenous Tat substrates produced in the cells during the time course of the experiment.

### The Number of TatA-GFP Foci Increases with an Enhanced Production of Tat Substrate

If the observed agglomeration of TatA-GFP is in fact linked to the formation of functional Tat translocases, over-production of a Tat substrate might have an influence on the number of TatA-GFP foci. As shown in Figure 3C, this was indeed the case. Upon co-expression of the Tat substrate TorA-mCherry, the number of bright dots increased considerably now appearing in various numbers and localizing predominantly to the cell boundaries (cf. Figure S4). Time-lapse pictures revealed that the majority of these dots are highly mobile often ending and fusing with each other at the cell poles (Movie S1). Incidentally, because these cells show an unimpaired export of the co-expressed TorA-mCherry to the periplasm, as indicated by the discrete halo-shaped distribution of the mCherry fluorescence (Figure 3C, inset), obviously our TatA-GFP fusion protein did not grossly interfere with the Tat transport catalyzed by the chromosomally encoded TatABC proteins. Moreover, co-expression of TorA-mCherry caused an increase in the number of TatA-GFP foci in spite of the fact that the expression level of TatA-GFP simultaneously decreased (Figure 2A, lanes 6 and 8), arguing against the possibility that the large fluorescent dots are merely aggregates of over-expressed TatA-GFP. Finally, TatA-GFP when actively promoting export of TorA-mCherry as sole *tatA* allele in the Δ*tatAE* strain (Figure 2D) also formed large dots (Figure 2E). This rules out the theoretical possibility that the TatA-GFP clusters were simply due to homo-oligomerization between TatA-GFP and endogenous TatA. Our results therefore suggest that the bright dots reflect active translocases that became visible through the association of TatA-GFP with the endogenous TatABC proteins. This conclusion is further supported by the finding illustrated in Figure 3D. Cells expressing the inactive KK-variant of TorA-mCherry, which as expected was retained in the cytosol (Figure 3D, inset), showed only the few foci seen in cells that do not produce any plasmid-encoded but only endogenous Tat substrates at native levels (compare Figure 3A and D).

**Figure 4:**
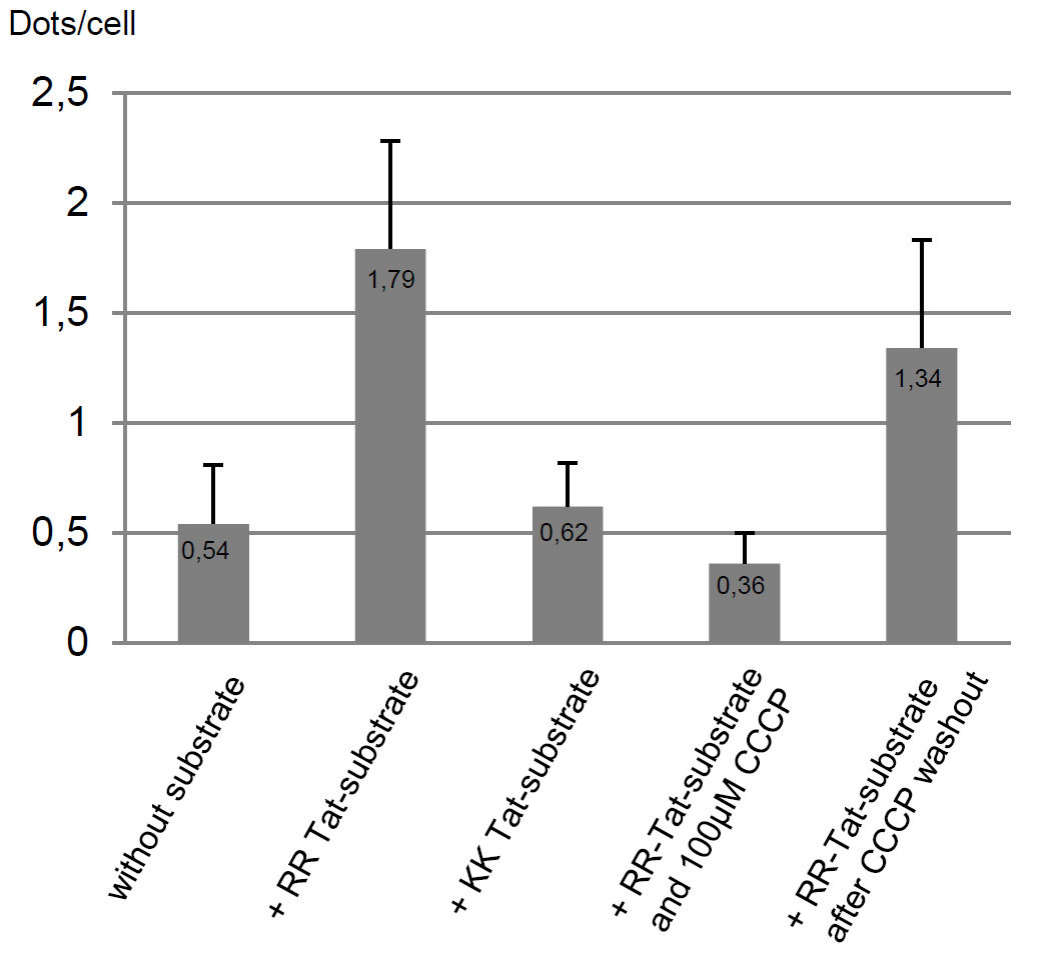
Quantification of TatA-GFP clusters. Using the cell image analysis software CellProfiler as detailed in Materials and Methods, the average number of bright dots per 150 to 500 cells was each counted. Indicated are mean values with the standard errors of the means. Quantified were cells as depicted in Figure 3A (without substrate), 3C and 5A (+ RR-Tat substrate), 3D (+ KK-Tat substrate), 5B (+ RR-Tat substrate and 100 µM CCCP), and 5D (+ RR-Tat substrate after CCCP washout).

To obtain a more quantitative evaluation of these results, the number of bright dots exceeding a given threshold intensity was quantified in 350 to 500 cells of each of the three cell populations depicted in Figure 3A, C, and D analyzing pictures from 4 to 6 independent experiments each. The data are summarized in Figure 4. Whereas cells that expressed TatA-GFP either alone or together with the inactive KK-variant of TorA-mCherry contained on an average 0.5 dots, co-expression of the active Tat substrate increased the number of dots more than three-fold. Hence there is a significant correlation between the production of functional Tat substrates to be exported by the *E. coli* cells and the number of bright foci originating from a clustering of TatA-GFP.

### Dependence on the Proton-Motive-Force Suggests TatA-GFP Clusters Highlight Functional Tat Translocases

**Figure 5:**
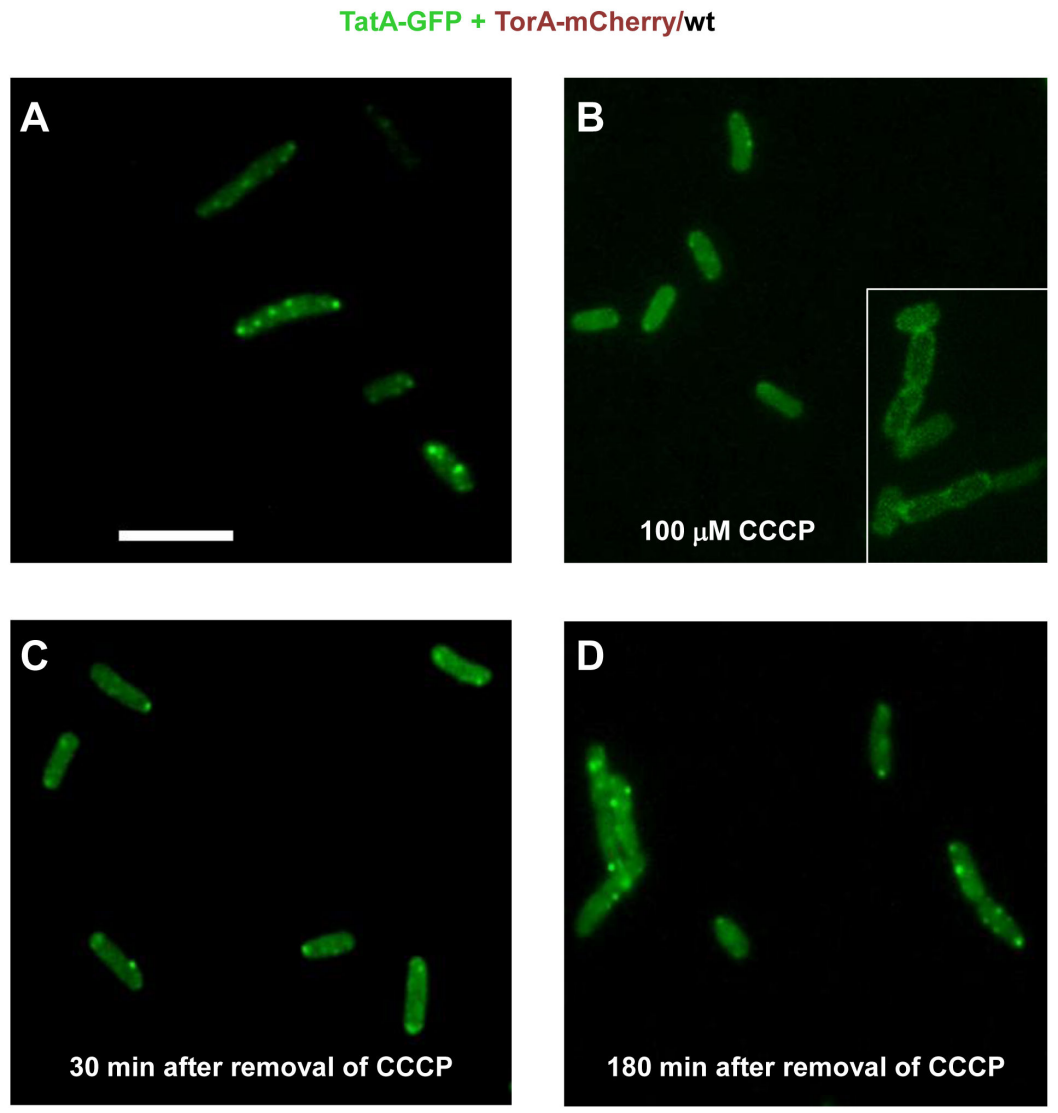
PMF-dependent clustering of TatA-GFP. (**A**) *E. coli* BL21(DE3) wild type cells (wt) co-expressing TatA-GFP from a pBAD33 vector (pPR1) and TorA-mCherry from a pET22 vector (pPJ3) show enhanced clustering of TatA-GFP as in Figure 3C. (**B**) A 30 min exposure of these cells to 100 µM of the protonophore CCCP impairs clustering in favor of a uniform distribution of TatA-GFP in the cell membrane (inset). (**C**–**D**) Removal of CCCP by washing the cells two times with fresh growth medium restores the clustering of TatA-GFP after 30 min and 180 min, respectively.

The Tat pathway requires energy from the proton-motive force (PMF) and published data indicate that specifically the recruitment of TatA oligomers to the TatBC receptor complex requires the transmembrane H^+^-gradient [23,52]. Therefore, if the observed clustering of TatA-GFP in fact reflects the formation of active translocases, it should be sensitive to dissipating the PMF of intact *E. coli* cells by the protonophore CCCP (carbonyl cyanide m-chlorophenyl-hydrazone). Figure 5A depicts an experimental situation identical to that of Figure 3C, in which the co-expression of TatA-GFP with the Tat substrate TorA-mCherry led to the appearance of numerous large and bright TatA-GFP foci. This pattern did not change when cells had been incubated for an additional 30 min in the presence of 0.1% DMSO used as the solvent for the treatment with CCCP (not shown). If, however, cells were incubated for about 30 min with 100 µM CCCP, the number of bright and clearly discernible dots visibly decreased (Figure 5B). Quantitation of about 300 cells and pictures taken from a representative number of parallel experiments revealed that after addition of 100 µM CCCP the number of bright TatA-GFP dots per cell decreased even below the average obtained for cells that did not synthesize any TorA-mCherry (Figure 4). In a number of CCCP-treated cells a clearly uniform re-distribution of TatA-GFP in the cell membrane as a consequence of dissipating the PMF became manifest (Figure 5B, inset). To investigate if the CCCP-mediated decrease in TatA-GFP clustering could be reversed, CCCP-treated cells were washed twice and then further incubated with CCCP-free LB medium prior to the inspection under the microscope. Figure 5C and D clearly illustrate the time-dependent re-appearance of the bright TatA-GFP dots, which was confirmed by quantitation (Figure 4). These results are fully consistent with the idea that the bright TatA-GFP foci reflect functional Tat translocases, which are jointly formed by the chromosomally encoded TatABC proteins and TatA-GFP complexes in a substrate- and PMF-dependent manner.

### Polar Clustering of TatB and Co-localization with TatA

**Figure 6:**
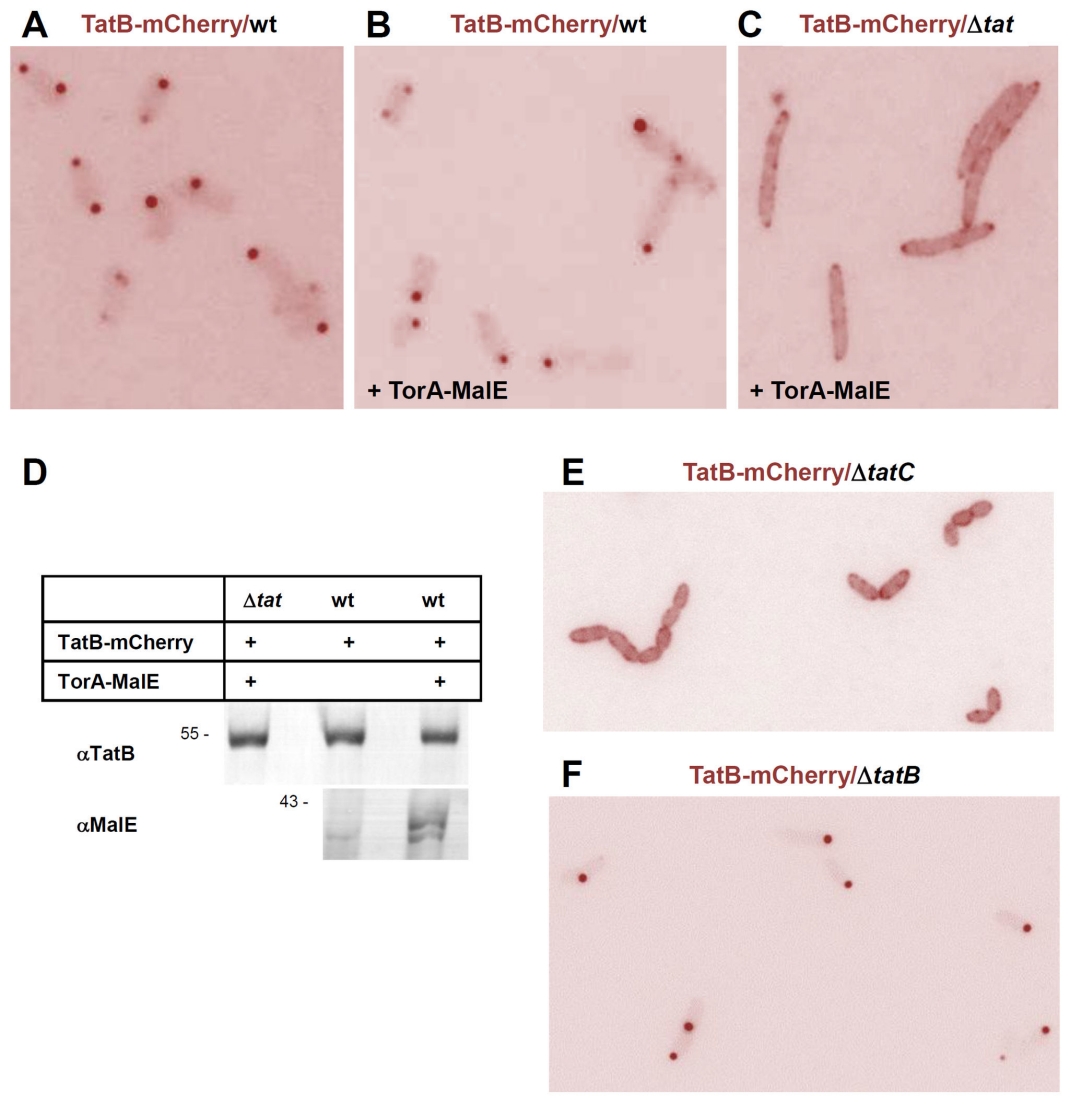
In the presence of TatA and TatC, TatB localizes almost exclusively to the cell poles. (**A**) *E. coli* BL21(DE3) wild type cells (wt) expressing TatB-mCherry from a pBAD33 vector (pPR2) following 2 h of induction with 0.1% arabinose. The fluorescence signal accumulates predominantly in polar foci with almost no staining of the cell bodies. (**B**) Co-expression of TatB-mCherry from a pBAD33 vector (pPR2) as in (A) and of the Tat substrate TorA-MalE335 from a pET22 vector (pPJ1) at non-induced levels. The additionally expressed Tat substrate does not change the staining pattern of TatB-mCherry. (**C**) Co-expression of TatB-mCherry and TorA-MalE335 in a BL21(DE3) Δ*tat* cells. Without TatA and TatC (Δ*tat*), TatB-mCherry is found dispersed in the cell periphery rendering the shape of the cells now more discernible against the background. (**D**) Equal expression levels of TatB-mCherry in cells depicted in (A–C). Whole cell proteins were precipitated with trichloroacetic acid and equivalent amounts were each subjected to SDS-PAGE. Immunoblots were decorated with antibodies against TatB and MalE as indicated. (**E**) Expression of TatB-mCherry in a Δ*tatC* strain (B1lK0). With TatC missing but native levels of TatB in the cells, TatB-mCherry is distributed evenly in the membrane as observed in a Δ*tatABC* strain (C). (**F**) When TatB-mCherry is expressed in a Δ*tatB* strain (BΦD) with endogenous TatC present, a polar localization of TatB-mCherry is observed as in a TatABC wild-type strain (A, B).

If this interpretation is correct, the TatA-GFP-labeled foci should also contain TatB and TatC. To address this question, we tagged TatB C-terminally with mCherry (pPR2) using the same pBAD expression vector as for TatA-GFP. The use of different fluorophore tags on TatA and TatB (GFP and mCherry, respectively) allowed following the localization of both proteins simultaneously in the same cells (see below). When TatB-mCherry, which was able to functionally replace TatB (Figure S3), was expressed together with the chromosomally encoded TatABC proteins, the fluorescent signals were restricted to one or two discrete and prominent foci that almost exclusively localized to the cell poles (Figure 6A). The mCherry fluorescence was virtually absent from the cell bodies and peripheries. A predominant polar localization of fluorescently labeled TatB variants had previously been reported also by others [48,49] and in our hands, was independent of the co-expression of the Tat substrate TorA-MalE335 (Figure 6B). However, TatB-mCherry stained cells in quite a different manner, if it was expressed in the absence of any wild-type TatABC protein (Figure 6C). In Δ*tatABC* mutant cells, TatB-mCherry was no longer selectively enriched in one or two polar dots but appeared much more spread out along the cell periphery yet in a coarse-grained pattern. As for TatA-GFP, this phenotype was independent of the co-expression of a Tat substrate (compare Figure 6C with Figure S2B). Because under the conditions depicted in Figure 6A–C, TatB-mCherry was expressed to the same level (Figure 6D), its polar concentration is unlikely to be an aggregation artifact. Moreover, polar clustering of TatB-mCherry was strictly dependent on the presence of both TatAC partners as it was not observed in a single *tatC* deletion strain (Figure 6E) but occurred in a *tatB* knockout strain expressing endogenous TatA and TatC (Figure 6F). Thus accumulation of TatB-mCherry at the cell poles strongly suggests the formation of TatABC complexes.

**Figure 7:**
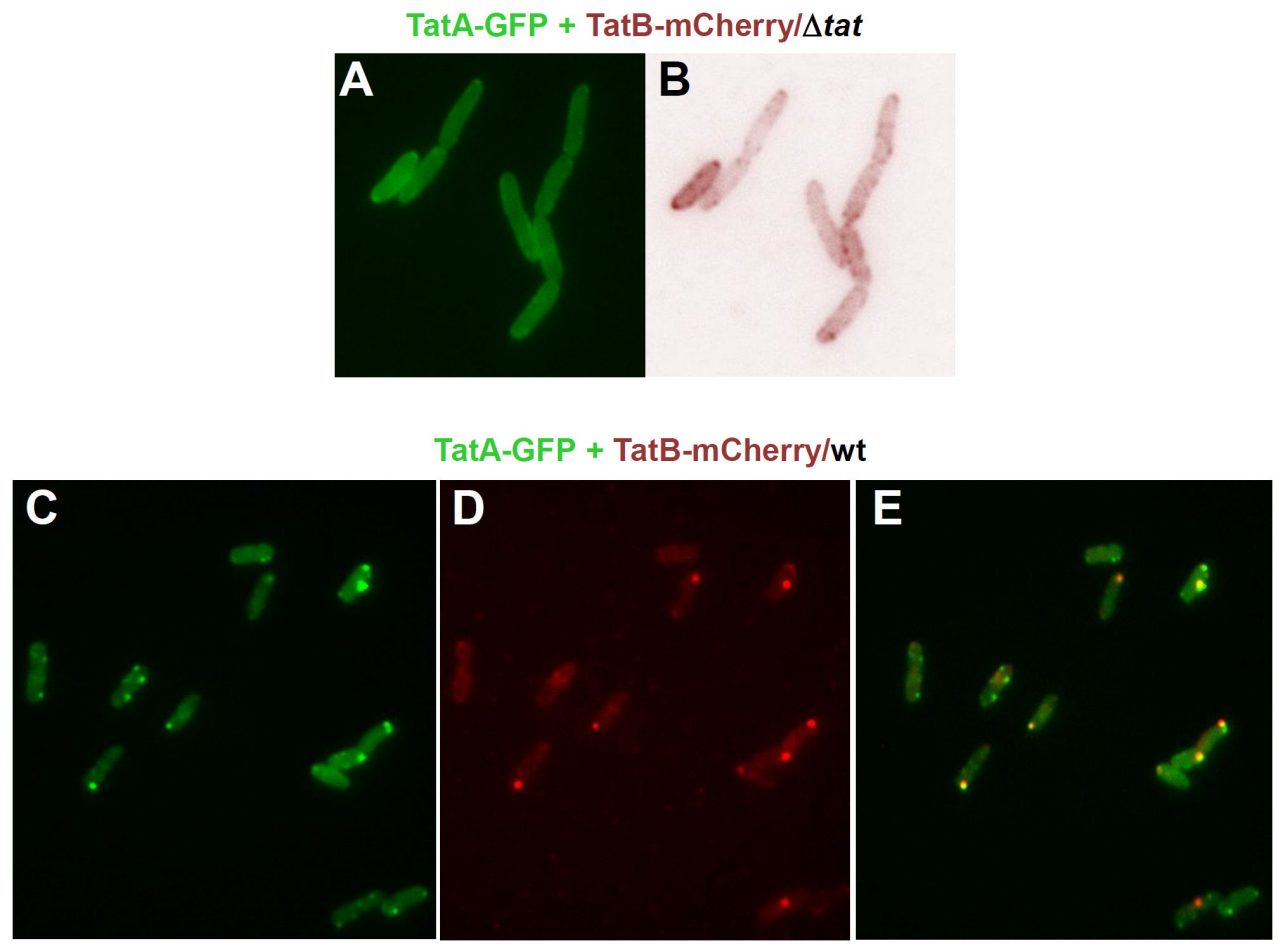
Co-localization of TatA and TatB. (**A**,**B**) TatA-GFP was expressed from a pQE-60 vector (pPR5) and TatB-mCherry from a pBAD33 vector (pPR2) in *E. coli* BL21(DE3) Δ*tat* cells (Δ*tat*). Cells were first induced with 0.1% arabinose for 1 h to start expression of TatB-mCherry and then for 1 h with 1 mM IPTG to additionally express TatA-GFP, before micrographs were taken. Without wild type TatABC, TatA-GFP (A) and TatB-mCherry (B) both localize uniformly to the cell periphery. (**C**–**E**) as (A,B) except that TatA-GFP and TatB-mCherry were co-expressed in *tatABC* wild type BL21(DE3) cells (wt). TatA-GFP (C) and TatB-mCherry (D) both form clusters, many of which coincide in the overlay (E).

In order to compare the distribution of TatA and TatB in the same cells, we simultaneously induced synthesis of TatA-GFP and TatB-mCherry in otherwise Tat deficient (Δ*tat*) cells (Figure 7A and B). Each fluorophore stained the same cells at the periphery with no single prominent foci appearing, which indicates a rather uniform distribution of TatA and TatB in the plasma membrane when TatC is absent and TatABC translocases cannot form. Rim-staining of cells by TatB-mCherry (Figure 7B) was more dotted than that by TatA-GFP (Figure 7A). Because this difference was also observed when both Tat fusions were expressed independently of each other (cf. Figures 3B and 6C), the more punctate pattern of TatB-mCherry seems to be a specific property of TatB, possibly reflecting a different degree of homo-oligomerization of both Tat subunits under these conditions. The picture changed again dramatically when both TatA-GFP and TatB-mCherry were co-expressed in wild-type *E. coli* cells, i.e. in the presence of TatC (Figure 7C–E). Now TatA-GFP and TatB-mCherry appeared predominantly as few bright dots, which in the case of TatA were more heterogeneous in intensity and intracellular localization than the more polarly enriched TatB foci. The brightest TatA and TatB dots, however, co-localized as demonstrated by an overlay of the GFP and mCherry pictures (Figure 7E and Movie S2). This finding lends strong support to the view that the GFP- and mCherry-stained dots mark the intracellular location of active TatABC translocases.

### Substrate-dependent Multimerization of TatC at the Poles

**Figure 8:**
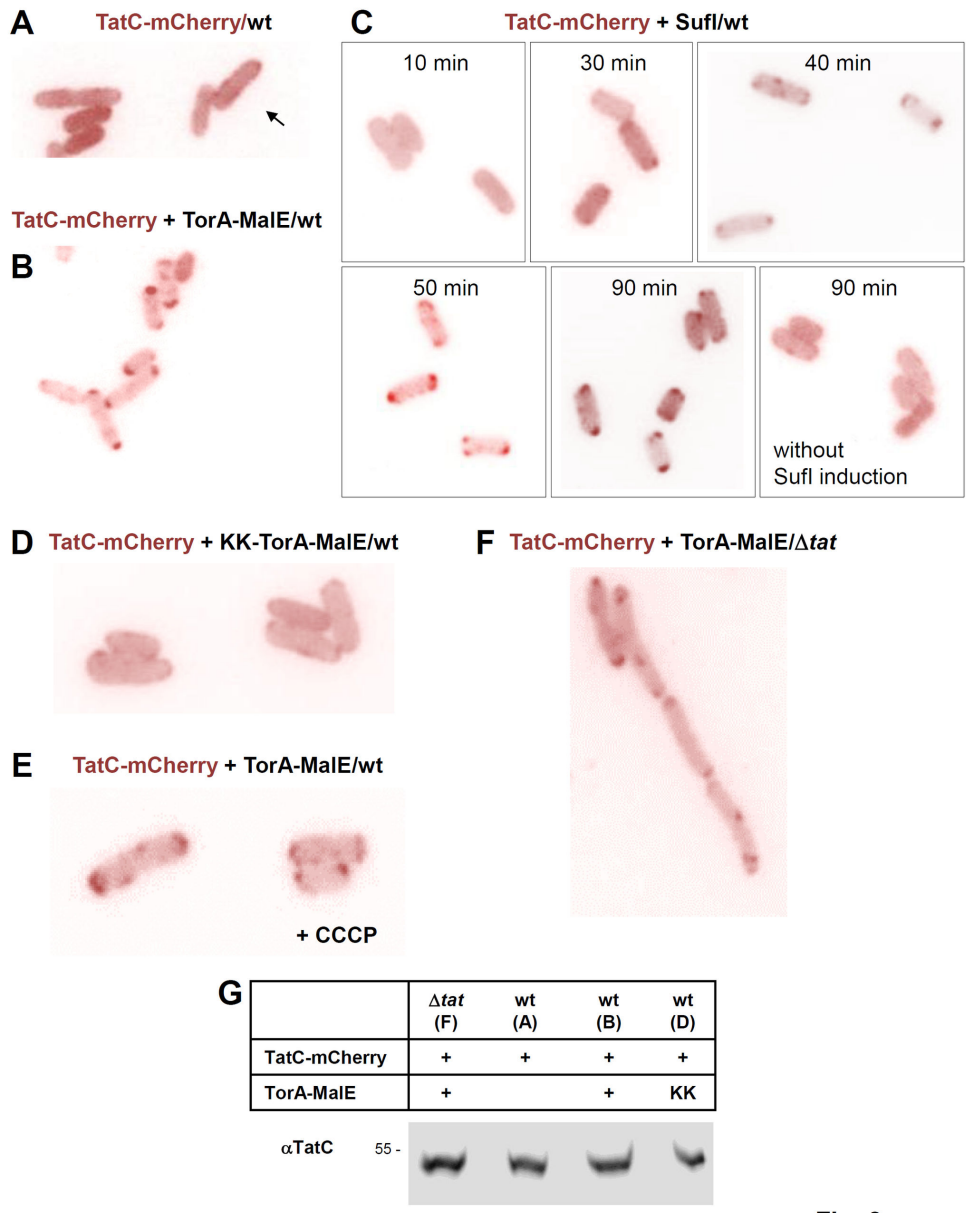
Expression of functional Tat substrates causes TatC to cluster at the cell poles. (**A**) *E. coli* BL21(DE3) wild type cells (wt) expressing TatC-mCherry from a pBAD33 vector (pPR3). Expression was induced by the addition of 0.1% arabinose for 2 h. Overall weak fluorescent signal that in some cells is concentrated at the cell periphery (arrow). (**B**) Co-expression of TatC-mCherry from a pBAD33 vector (pPR3) as in (A) and of the Tat substrate TorA-MalE335 from a pET22 vector (pPJ1) at non-induced levels. The additionally expressed Tat substrate causes a pronounced clustering of TatC-mCherry at the cell poles. (**C**) Co-expression of TatC-mCherry from a pBAD33 vector (pPR3) as in (A) and of the natural Tat substrate SufI from a tightly controlled pASK-IBA33plus vector (pPJ9) by the subsequent addition of 50 ng/ml anhydro-tetracycline. Samples were taken at the indicated times and inspected under the microscope. Appearance of polar clusters starts after 30 min with a maximum reached after 50 min. No clustering of TatC-mCherry occurs without induction of SufI. (**D**) as in (B) expressing the non-functional KK-version of TorA-MalE335 from plasmid pPJ2. This does not lead to re-localization and clustering of TatC-mCherry at the poles. (**E**) as in (B) except that after inducing synthesis of TatC-mCherry and TorA-MalE335, 100 µM CCCP was added to the growth medium 30 min before cells were microscoped. Dissipation of the PMF does not interfere with the TorA-MalE335-dependent agglomeration of TatC-mCherry at the cell poles. (**F**) as in (B) except that TatC-mCherry and TorA-MalE335 were expressed in BL21(DE3) Δ*tat* cells (Δ*tat*). The TorA-MalE335-mediated clustering of TatC-mCherry at the cell poles is also independent of TatA and TatB. (**G**) Equal expression levels of TatC-mCherry in the cells depicted in the indicated panels. After 2 h of induction, whole cell proteins were precipitated with trichloroacetic acid and equivalent amounts were subjected to SDS-PAGE. Immunoblots were decorated with antibodies against TatC.

Finally we wished to track the localization of TatC in live *E. coli* cells using the same experimental approach (Figure 8). When a C-terminal fusion between TatC and mCherry, which could functionally replace TatC (Figure S3), was expressed in wild-type cells, the staining pattern was rather diffuse, occasionally showing the formation of smaller clusters or accumulation at the cell periphery (Figure 8A, arrow). The diffuse picture could be explained by an overall lower fluorescence of GFP-type fluorophores when attached to TatC as previously recognized by others [48,49]. By contrast, co-expression of the Tat substrate TorA-MalE335 led to a massive concentration of TatC-mCherry at both poles of virtually every cell (Figure 8B). The appearance of the TatC-mCherry complexes at the cell poles was less spherical than those of TatB-mCherry (cf. Figure 6A, B) but more oval or often even cap-like (Figure 8B). These cap-like structures could result from the fusion of two or more adjacent spherical dots (as illustrated below in panels C, E, F) and were similarly found in previous studies [48,49]. To demonstrate that the polar localization of TatC was truly substrate-dependent, we performed a time-course experiment, in which TatC-mCherry-expressing *E. coli* cells were inspected under the microscope at different times after inducing the synthesis of the natural Tat substrate SufI, whose gene had been cloned under the control of a *tet* promoter (Figure 8C). The first polar TatC-mCherry dots appeared about 30 min after addition of the inducer anhydro-tetracycline and became fully prominent after 50–90 min, whereas the control cells grown in the absence of the inducer showed the same blurred distribution of stain as in the beginning of this experiment. The polar accumulation of TatC-mCherry was strictly dependent on a functional Tat substrate because it was not obtained in cells co-expressing the KK-variant of TorA-MalE335 (Figure 8D). In contrast, dissipation of the PMF did not prevent the substrate-mediated accumulation of TatC-mCherry at the poles (Figure 8E). This is in full accordance with the PMF-independent interactions between substrate and TatC previously established [34,40,53]. Quite remarkably, the Tat substrate TorA-MalE335 led to a massive concentration of TatC at the cell poles, even in the absence of TatA and TatB (Figure 8F), suggesting that a concentration of TatC at the cell poles is primarily caused by the presence of Tat substrate and obviously requires neither TatB nor TatA. Accordingly, no polar foci were observed in a Δ*tatABC* strain if no Tat substrate was co-expressed (Figure S2C). Polar localization of TatC was unrelated to the expression level of TatC-mCherry (Figure 8G) and thus complements the data obtained for the TatA and TatB fusions, strongly suggesting that *in vivo*, active Tat translocases accumulate on demand preferentially at the cell poles.

## Discussion

We demonstrate here that the TatABC subunits of live *E. coli* cells when encountering active Tat substrates cluster to form large functional Tat translocase complexes. The underlying experimental strategy was to express a fluorophore-tagged Tat subunit in addition to its chromosomally expressed counterpart. In this way, a Tat translocase could form from the native TatABC proteins and be visualized via the association of a fluorescent Tat variant. The validity of this strategy is indicated by the findings that clustering of TatA and TatB in fact required the presence of all three Tat subunits, and that the TatA-GFP clusters disappeared upon dissipating the PMF. Both interdependences would not exist if the fluorescent dots merely resulted from the aggregation of inactive TatA-GFP and TatB-mCherry fusion proteins, respectively. The notion that the fluorophore-tagged Tat proteins highlighted the formation of functional TatABC translocation sites is reinforced by the finding that clustering of TatA-GFP and TatC-mCherry was enhanced upon co-expression of Tat substrates with a functional RR-signal peptide. As expected for functional Tat translocases, TatA and TatB co-localized to the same fluorescent foci in cells that simultaneously expressed TatA-GFP and TatB-mCherry. In further support of the physiological relevance of our findings, we demonstrate that TatA-GFP actively participated in the translocation of RR-precursors, when it was the only TatA species in the cell, and that it did not interfere with Tat transport, when it was expressed on top of the chromosomally encoded TatA. To furthermore rule out that the GFP moiety led to artificial results by extensively impairing the function of TatA, we performed parallel experiments with TatA that was C-terminally labeled with a small tetracysteine tag binding the FlAsH reagent but basically obtained the same results (not shown). Hence the sum of these findings strongly supports the view that the fluorescent complexes represent functional Tat translocases.

Using a similar approach, yet expressing a TatA-YFP construct from the native *tatA* promoter, Leake et al. estimated that each *E. coli* cell contains 15 ± 9 TatA complexes [51]. Taking into account that this value was obtained by extrapolating the number of spots within the focal plane of the microscope to that of the whole cell surface, it seems to correlate reasonably well with the 0.5 dots that we determined on an average for each cell that had not been stimulated by any co-expression of Tat substrates. As to the size and stoichiometry of the fluorescently labeled Tat translocases, our data do not allow detailed conclusions. Using photobleaching, Leake et al. calculated a mean number of 25 TatA molecules in their prominent fluorescent dots [51]. In Gram-negative bacteria and chloroplasts, a functional TatBC receptor complex was estimated to be composed of seven to eight TatB: TatC dimers [41–43]. Celedon and Cline demonstrated that about 26 TatA monomers are required for the transport of each Tat substrate molecule into the thylakoids of pea chloroplasts and that each TatB: TatC protomer of the presumably octameric TatBC receptor complex binds one precursor molecule each [41]. This would amount to a maximal occupancy with over 200 TatA monomers and a molecular mass of about 2.2 MDa for an active Tat translocase. On the contrary, single particle electron microscopy of TatBC complexes isolated from *E. coli* showed only two molecules of Tat substrate bound to a probably heptameric TatBC complex [43] raising the possibility that functional Tat translocation complexes might display a considerable variation in their actual sizes.

In the experiments described here, most substrate-induced TatA-GFP foci were mobile with a certain tendency to move to the cell poles. The TatB-mCherry complexes co-localized to a large extent with the TatA-GFP clusters and both TatA and TatB fusions only accumulated at the poles in the presence of all Tat subunits. In full accordance with those polar TatAB foci representing Tat translocation sites, also TatC-mCherry clustered at the poles when challenged by a Tat substrate. Different from TatA and TatB, however, this substrate-mediated concentration of TatC at the poles did not require the presence of the other Tat subunits. This is reminiscent of previous findings indicating the productive interaction between RR-signal peptides and TatC even in the absence of TatB and TatA [34,40]. Whilst those data underline the significance of the initial binding of Tat substrates to TatC, we have now established that the re-organization of the individual Tat subunits from a rather uniform distribution in the plasma membrane to the cell poles is triggered by the production of Tat substrates.

These data raise the question why the *E. coli* Tat translocase would preferentially assemble, and presumably also operate, at the cell poles. It has been noted that the large membrane curvature at the poles of rod-shaped bacteria seems to evoke lipid domains with a conspicuous accumulation of cardiolipin [54,55]. Such cardiolipin-rich micro domains seem to be determinants for the polar localization of certain membrane proteins [54] and might conceivably also be required to position, or even help to assemble, huge protein complexes such as the Tat translocase. Consistent with a cardiolipin-containing boundary domain of Tat translocase complexes would be the finding that the export of a Tat substrate is severely retarded in *E. coli* cells that had been depleted of this anionic phospholipid [56]. The relevance of this finding with respect to polar export sites for Tat substrates is, however, less evident as these authors found an impaired Tat export also upon depletion of phosphatidyl-ethanolamine. Another conceivable reason for the Tat transport occurring at the cell poles could be the presumed lipid-packing defects caused by the high positive curvature of the outer leaflet of the *E. coli* plasma membrane. It has been proposed that the membrane crossing of folded Tat substrates could either involve a flipping of the amphipathic helices of connected TatA molecules into the lipid bilayer (trapdoor mechanism [23]:) or a general destabilization of the bilayer by TatA (membrane weakening hypothesis [5,25,26]:). Both as yet hypothetical events might be facilitated by lipid-packing defects at the cell poles. Moreover, PspA is a member of a family of so called phage-shock proteins that have been shown to combat proton leakage across the bacterial plasma membrane caused by different sorts of membrane stress [57]. PspA, which was found to stimulate Tat transport [58–60], has recently been shown to locate close to TatA and thereby could help minimizing any proton loss during the Tat translocation event [61]. Of note, PspA of *Yersinia enterocolitica* was recently found to re-locate to membrane clusters close to the cell poles when induced [62]. In any event, our data clearly show that the Tat complex can also assemble at the lateral sides of the cell, such that the pole does not appear to be an absolute prerequisite for efficient export.

Whereas the property of the TatABC subunits to form oligomeric assemblies following detergent solubilization from cellular membranes had been appreciated since long (comprehensively reviewed in 2, the data presented here now demonstrate that TatABC complexes dynamically form in living cells in response to the supply of newly synthesized Tat substrates, with TatC functioning as the primary binding site and TatA and TatB following to finally lead to efficient transport into the periplasm.

## Materials and Methods

### DNA Manipulations

PCRs were performed using *Pfu* polymerase (Fermentas). The PCR products were extracted from agarose gels by use of a Qiagen gel extraction kit. Dephosphorylations were carried out with antarctic phosphatase NEB (New England Biolabs), DNA purifications were performed using the PCR-purification kit (Qiagen). All restriction enzymes were purchased from New England Biolabs. Plasmid constructs were all verified by DNA sequencing.

### PLASMIDS

To construct plasmid pPR1 (pBAD33SD/TatA-GFP), *tatA* was amplified via PCR using plasmid p8737 (pET22b+/TatABCD) as template [63]) and the primers 5´-SacI TatA Primer and 3´-BglII TatA Primer (table S1). In a second PCR reaction, DNA encoding the highly fluorescent GFP variant *GFPmut1* was amplified using the primers 5-BlpI GfpMut1 and 3-XbaI GfpMut1 and plasmid pSG1164 [64] as template. PCR fragments were each digested with the corresponding primer-encoded restriction enzymes and ligated via a 36 bp linker sequence generated by the two phosphorylated, partially complementary oligodeoxynucleotides P-5-SufI-Linker and P-3-SufI-Linker. Prior to ligation, both oligonucleotides were hybridized for 5 min at room temperature. The resulting sticky 5´-BglII and 3´-BlpI ends allowed the ligation of the *tatA* and *GFPMut1* PCR products. The newly formed TatA-linker-GFP DNA fragment was purified and digested with SacI and XbaI and ligated into the pBAD33 vector, which had been digested with the same restriction enzymes followed by dephosphorylation. To introduce a Shine-Dalgarno (SD) sequence, the resulting pBAD33/TatA-GFP vector was linearized with SacI upstream of *tatA* and dephosphorylated. Using the strategy described above for the insertion of the linker sequence and the oligodeoxynucleotides P-5´-SacI SD pBAD und P-3´-SacI SD pBAD, a stretch of DNA encoding the SD of vector pET22b+ was generated with SacI restriction sites at both ends and ligated into pBAD33/TatA-GFP to give the final construct pBAD33SD/TatA-GFP (pPR1).

To obtain plasmid pPR2 (pBAD33SD/TatB-mCherry), plasmid pBAD33SD/TatA-GFP was digested with BglII and XbaI to remove the GFP-encoding fragment. It was then dephosphorylated and ligated with a cleaved and purified PCR fragment encoding mCherry, which had been obtained by PCR using plasmid pRSET-mCherry [65] as template and the primers 5-BglII mCherry and 3´-XbaI C-mCherry. The resulting plasmid (pBAD33SD/TatA-mCherry) was digested with SacI and BglII to excise the *tatA* gene, dephosphorylated and ligated with a second purified PCR fragment encoding TatB. This fragment had been obtained using the primers 5´-SacI TatB Primer and 3´-BglII TatB Primer and plasmid p8737 as template. An SD-sequence was re-introduced into the resulting plasmid pBAD33/TatB-mCherry as described for plasmid pPR1.

Plasmid pPR3 (pBAD33SD/TatC-mCherry) was constructed by cleaving the vector pBAD33/TatB-mCherry with SacI and BglII, to remove *tatB* and replace it by *tatC*. The TatC-encoding DNA was amplified via PCR using the primers 5´-SacI TatC Primer and 3´-BglII TatC Primer and plasmid p8737 as template. A SD-sequence was introduced as described for pPR1

Plasmid pPR4 (pBAD33SD/TatA) was constructed by cutting out the GFP fragment from pBAD33SD/TatA-GFP with BglII and XbaI. The resulting sticky ends were filled up with Klenow fragment, phosphorylated and ligated. This was done by mixing 2 µL vector DNA (~30ng/µL), 1 µL 10x Fermentas T4 DNA ligase buffer, 4 µL H_2_O, 2 µL dNTP-mix (10mM each) and 1 µL Klenow enzyme (NEB) and incubation for 20 min at room temperature. Subsequently 1 µL T4 polynucleotide kinase (NEB) was added to the mixture and incubated for 1 h at 37° C followed by a 1 h ligation reaction with 1 µL T4 Ligase (Fermentas). This procedure generated an in frame TAG stop codon causing the extension of TatA by two amino acids compared to the original sequence.

To generate plasmid pPR5 (pQE-60/TatA-GFP), the pQE-60 vector was cut with NcoI and BamHI and ligated with a TatA-encoding fragment that was obtained by PCR using p8737 as template and the primers NcoI-TatA5´-Primer and BamHI-TatA3´-Primer. In a second step, a linker was added at the 3´-end of TatA as detailed for the construction of pPR1 using the oligodeoxynucleotides P-5´-Linker and P-3´-Linker. In a third step, the GFP-encoding sequence was inserted into the plasmid downstream of the linker. The corresponding PCR fragment was obtained using the primers NheI-cYfp 3´-Primer and 5-BlpI GfpMut1 and pSG1164 as template.

For creating plasmid pPR7 (pASK-IBA33plus/TorA-mCherry), the DNA encoding TorA-mCherry was amplified by PCR from plasmid pPJ3 (pET22b+/TorA-mCherry) [21] with the primers 5-pASK TorA-mcherry BSAI and 3-pASK TorA-mcherry BSAI. The resulting DNA fragment and the empty pASK-IBA33plus vector were digested with BsaI and ligated.

To increase the signal to noise ratio of periplasmically located TorA-mCherry, in particular in derivatives of *E. coli* strain MC4100, an SsrA-tagged version of TorA-mCherry was created allowing degradation of cytosolically retained TorA-mCherry [60]. To obtain the SsrA-tagged version of TorA-mCherry, plasmid pPJ3 (pET22b+/TorA-mCherry) [21] was linearized with XhoI, desphophorylated and ligated with the hybridized oligodeoxynucleotides 5-P-XhoI SsrA-Tag and 3-P-XhoI SsrA-Tag encoding the SsrA sequence and possessing sticky XhoI ends. The resulting plasmid is pPR6 (pET22b+/TorA-mCherry-SsrA). Plasmid pPR8 (pASK-IBA33plus/TorA-mCherry-SsrA) was constructed by amplifying via PCR the TorA-mCherry-SsrA-encoding sequence from plasmid pPR6 using the primers 5-pASKIBA33 TorA-mCherry-SSrA and 3-pASKIBA33 TorA-mCherry-SSrA. The PCR product and the empty pASK-IBA33plus vector were digested with BsaI and ligated.

Plasmids pPJ5 (pET22b+/TorA(KK)-mCherry) [21], pPJ9 (pASK-IBA33plus/SufI) [40], pPJ1 (pET22b+/TorA-MalE335) [21] and pPJ2 (pET22b+/TorA(KK)-MalE335) [32] have been described previously.

### Western Blotting and Fractionation

Whole cell proteins were precipitated with 5% trichloroacetic acid (TCA) and loaded onto 12% SDS-polyacrylamide gels. Polyclonal antibodies against TatA, TatB, TatC [34,63] and MalE were used in 1:5 000 dilutions, those against SufI in a 1:8 000 dilution. For developing the TatA, TatB, TatC and MalE-blots, goat anti-rabbit IgG coupled to alkaline phosphatase (Sigma) were used (1:20 000). Detection was performed using nitro-blue tetrazolium chloride/5-Bromo-4-chloro-3-indoyl phosphate (Roche Applied Science) following the manufacturer’s instructions. The SufI blot was developed using goat anti-rabbit IgG coupled to horseradish peroxidase (Kirkegaard & Perry Laboratories) (1:20 000). Detection was performed with ECL Western Blotting Detection Reagent (GE Heathcare, Amersham) following the manufacturer’s instructions.

For cell-fractionation, 20 mL LB-medium was inoculated with 200 µL overnight culture and induced at an OD_600_ = 0.3 with 0.1% L-arabinose for 1.5 h followed by an induction with anhydro-tetracycline (50 ng/mL) for an additional 1.5 h at 37° C and 180 rpm. Cells were pelleted, washed with ice-cold 30 mM Tris-HCl, pH8 and resuspended in 2 mL sucrose-solution (20% sucrose, 30 mM Tris-HCl, pH 8). Cells were gently treated with 1 mL 0.05 mg/mL lysozyme, 0.1 M EDTA for 5 min on ice and afterwards pelleted by centrifugation at 17 600 x g for 15 min at 4° C. One third of the supernatant was precipitated with 5% TCA and taken as the periplasmic fraction (P). The whole pellet was washed with sucrose-solution, resuspended in 1 mL 30 mM Tris-HCl, pH 8, precipitated with 5% TCA and taken as the cytoplasm/membrane fraction (C/M). The periplasmic fraction was resuspended in 500 µL, the cytoplasm/membrane fraction in 1 mL loading buffer. For SDS-PAGE, 30 µL of the P fraction and 5 µL of the C/M fraction (diluted 1:10) were loaded onto an 8% SDS gel.

### Bacterial Strains and Microscopy

*E. coli* strains MC4100 [66], DADE [67], JARV16 [13], BΦD [13], B1LK0 [68], BL21(DE3) (Novagen), BL21(DE3) Δ*tat* (Δ*tatABC*) [40] were grown aerobically at 37° C and 180 rpm in LB-media. Media supplements were used at the following final concentrations: ampicillin (50 µg/mL), Chloramphenicol (35 µg/mL), apramycin (50 µg/mL), IPTG (1 mM), L-arabinose (0.1%, unless stated otherwise), anhydro-tetracycline (50 ng/mL). For growth tests on LB Plates with 2% SDS and 0.1% arabinose, cells were grown in LB media with appropriate antibiotics to OD_600_ of 0.1, serially diluted and then dropped onto the agar plate. After 16 h of incubation at 37° C, growth was documented. For cell-imaging, cells were grown in LB media with appropriate antibiotics to OD_600_ of 0.5, induced as detailed in the figure legends and fixed on thin-coated agarose slides (1% agarose dissolved in H_2_O) and examined by phase contrast and fluorescence microscopy. Images were taken with an Olympus BX 51 microscope with a X-cite 120 light source and the Olympus software Cell-F at 1000-fold magnification with a numerical aperture of 1.4 and a Cy3 (for mCherry) and EGFP HC (for GFP) fluorescence filter set. Images were taken with a charge-coupled device camera (F-View, Olympus, Hamburg, Germany). The digital images were processed using the program ImageJ (http://rsbweb.nih.gov/ij/) and analyzed by using the cell image analysis software CellProfiler (Broad Institute). The analysis was automated by the development of Cell Profiles pipelines (available on request), allowing the processing of large numbers of images and recording all values in databases. For each growth condition, values obtained from 150-500 cells were compiled.

## Supporting Information

Figure S1 **Localization of TorA-mCherry in individual tat deletion strains.**
(**A**) Fluorescent micrographs of *E. coli tatABC* wild type (wt) cells of strain MC4100. TorA-mCherry was expressed from a pASK-IBA33plus vector (pPR7) after induction with 50 ng/ml anhydro-tetracycline for 2 h. Rim staining can be observed in most of the cells. The insets show cells taken from different micrographs. (**B**–**D**) Expression of TorA-mCherry in the MC4100 deletion versions JARV16 (Δ*tatAE*), BΦD (Δ*tatB*), B1LK0 (Δ*tatC*). All three individual Tat deletion mutants show the typical cell chains and retain the fluorescent signal of TorA-mCherry in the cytosol. Presumed binding of TorA-mCherry to the TatBC-complex in the Δ*tatAE* cells is therefore not sufficient to yield cellular rim staining, which was observed only in cells with functional Tat translocases.
(TIF)

Figure S2 **Localization of TatA-GFP, TatB-mCherry and TatC-mCherry in a BL21(DE3) Δ*tat*strain without simultaneous expression of an extra Tat substrate**.
Fluorescent micrographs of *E. coli* BL21(DE3) Δ*tatABC* cells erxpressing TatA-GFP (A), TatB-mCherry (B) and TatC-mCherry (C) each from a pBAD33 vector. TatA-GFP (A) and TatB-mCherry (B) show the same peripheral accumulation as when co-expressed with a plasmid-encoded Tat substrate in a *tatABC* deletion mutant (cf. Figures 3B and 6C). TatC-mCherry (C) does not accumulate in polar foci but shows the same diffuse distribution throughout the cell bodies seen whenever no additional Tat substrate was expressed (cf. Figure 8 A,C,D).
(TIF)

Figure S3 **Restoration of growth of *tatB* and ***tatC*** mutant cells on 2% SDS by TatB-mCherry and TatC-mCherry fusions.**
TatB, TatC, TatB-mCherry (TatB-mCh) and TatC-mCherry (TatC-mCh) were each expressed from pBAD33 vectors in TatABC wild-type, Δ*tatC* mutant (strain B1lK0), and Δ*tatB* mutant (strain BΦD) cells, as indicated. Cells grown in LB liquid media were adjusted to an OD_600_ of 0.1 and serially diluted and 5µl of each dilution was applied to agar plates containing 2% SDS and 0.1% arabinose.
(TIF)

Figure S4 **Localization of TatA-GFP in a TatABC wild-type strain co-expressing TorA-mCherry.**
(**A**) Same picture as shown in Figure 3C. (**B**) Phase contrast image of the same cells.
(TIF)

Movie S1 **Dynamics of TatA-GFP clusters.**
BL21(DE3) cells expressing TatA-GFP from plasmid pPR1 were grown and induced as described in Material and Methods. Fluorescence microscopy was performed using a Zeiss Axio Observer Z1 microscope and a 100x objective with a numerical aperture (NA) of 1.45, using a TIRF setup from Visitron (Munich, Germany). The movie was recorded with an Evolve EM-CCD camera (Photometrix) and processed with Metamorph 6.3 (Universal Imaging Corp.) and ImageJ 1.44 software. GFPmut1 was excited by a laser of 488 nm wavelength. The movie (5 frames/sec) shows the real-time movement of TatA-GFP in two cells. At their distal poles, both cells show a dynamic appearance and localization of TatA-GFP clusters including apparent fusions of two or more clusters. At the presumed division site of the left cell a new cluster is formed from several foci that originate from positions in the middle of the cell.
(AVI)

Movie S2 **Mobile fluorescent clusters simultaneously containing TatA and TatB.**
Same cells as shown in Figure 7 C–E simultaneously expressing TatA-GFP and TatB-mCherry in a *tatABC* wild-type background. The simultaneously recorded GFP- and mCherry-fluorescence is presented in constantly alternating pictures. Note the one major dot originating from the right side of the right cell first moving upwards and subsequently downwards to finally fuse with a dot at the lower pole. Movements are fully congruent for both stains.
(AVI)

Table S1 Primers used to construct the indicated plasmids.
(DOCX)
