# Supplementary figures and images for: Substrate-Dependent Assembly of the Tat Translocase as Observed in Live Escherichia coli Cells
Source: PLoS One. 2013 Aug 2;8(8):e69488. doi: 10.1371/journal.pone.0069488 (PMC3732296; doi:10.1371/journal.pone.0069488)

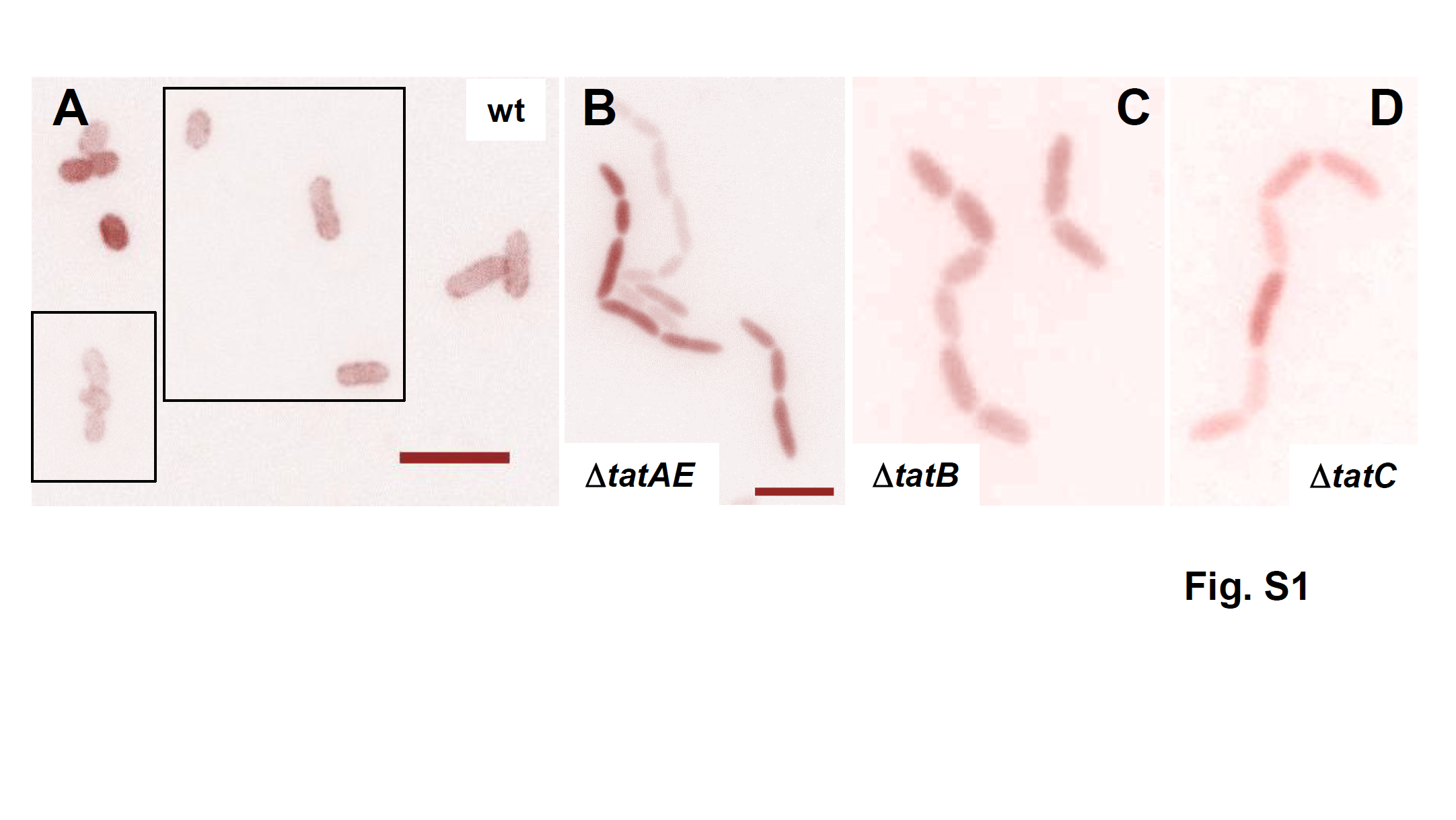

Supplement: Figure S1 — Localization of TorA-mCherry in individual tat deletion strains. (A) Fluorescent micrographs of E. coli tatABC wild type (wt) cells of strain MC4100. TorA-mCherry was expressed from a pASK-IBA33plus vector (pPR7) after induction with 50 ng/ml anhydro-tetracycline for 2 h. Rim staining can be observed in most of the cells. The insets show cells taken from different micrographs. (B–D) Expression of TorA-mCherry in the MC4100 deletion versions JARV16 (ΔtatAE), BΦD (ΔtatB), B1LK0 (ΔtatC). All three individual Tat deletion mutants show the typical cell chains and retain the fluorescent signal of TorA-mCherry in the cytosol. Presumed binding of TorA-mCherry to the TatBC-complex in the ΔtatAE cells is therefore not sufficient to yield cellular rim staining, which was observed only in cells with functional Tat translocases. (TIF) [file pone.0069488.s001.tif]

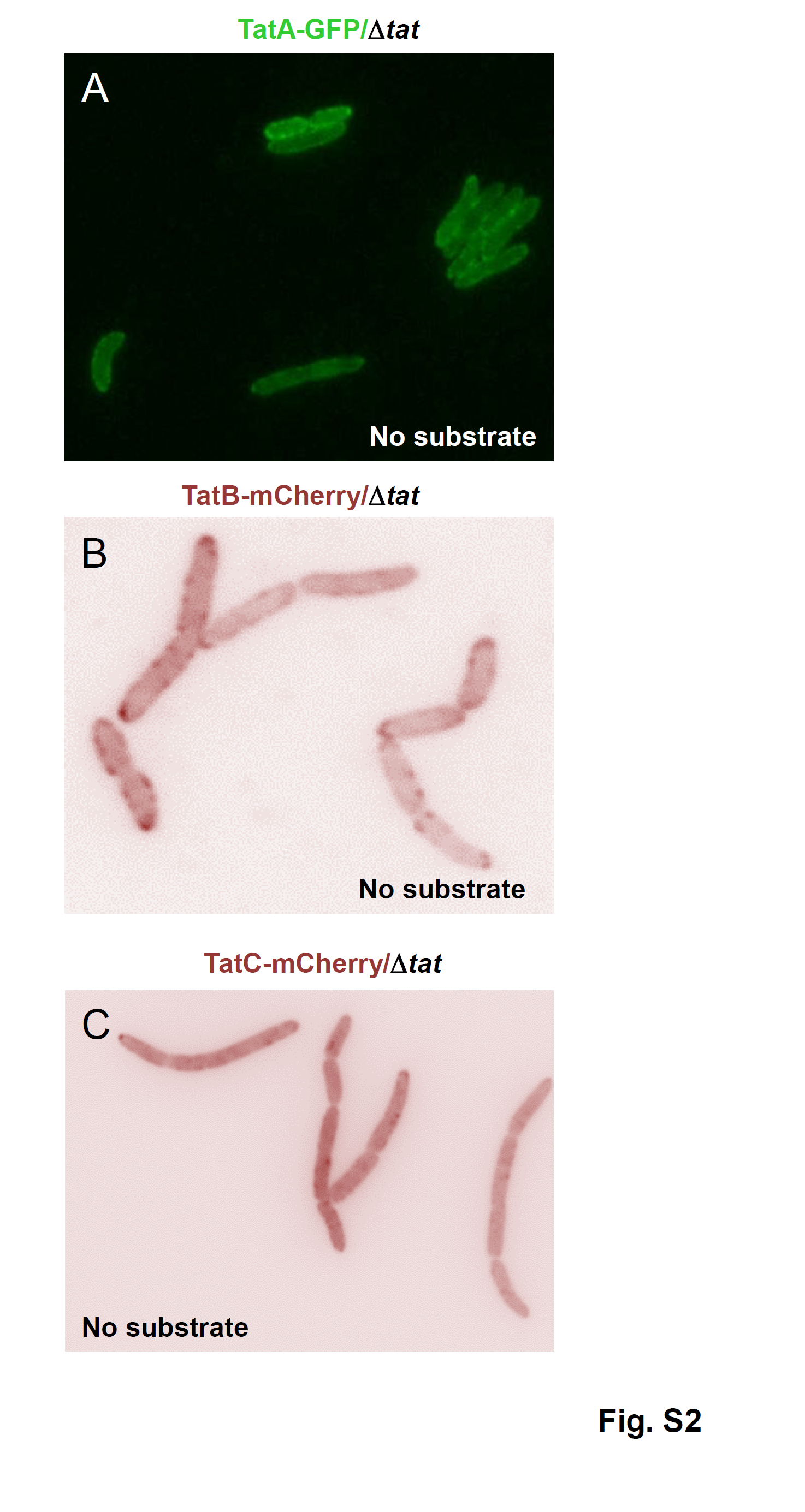

Supplement: Figure S2 — Localization of TatA-GFP, TatB-mCherry and TatC-mCherry in a BL21(DE3) Δtatstrain without simultaneous expression of an extra Tat substrate. Fluorescent micrographs of E. coli BL21(DE3) ΔtatABC cells erxpressing TatA-GFP (A), TatB-mCherry (B) and TatC-mCherry (C) each from a pBAD33 vector. TatA-GFP (A) and TatB-mCherry (B) show the same peripheral accumulation as when co-expressed with a plasmid-encoded Tat substrate in a tatABC deletion mutant (cf. Figures 3B and 6C). TatC-mCherry (C) does not accumulate in polar foci but shows the same diffuse distribution throughout the cell bodies seen whenever no additional Tat substrate was expressed (cf. Figure 8 A,C,D). (TIF) [file pone.0069488.s002.tif]

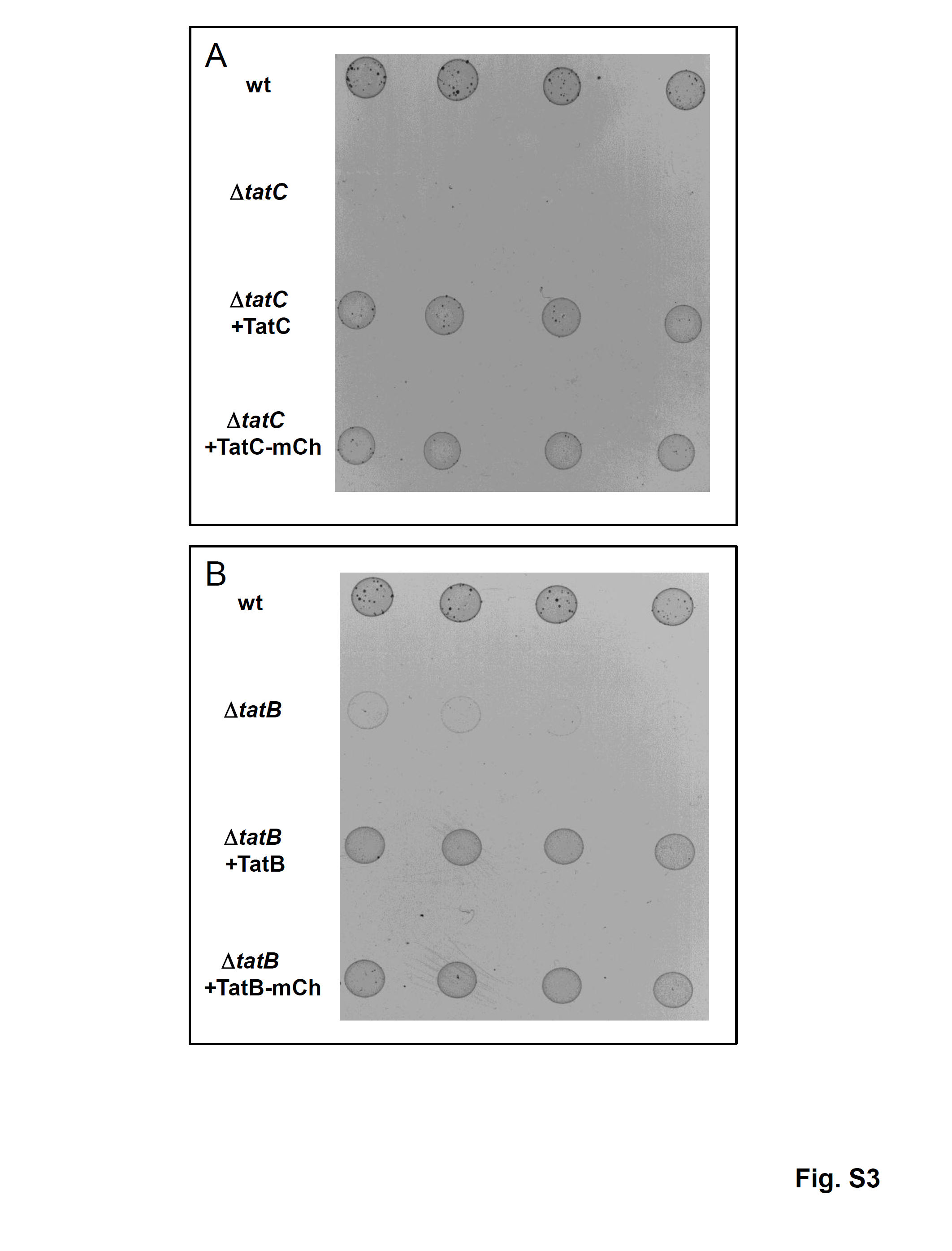

Supplement: Figure S3 — Restoration of growth of tatB and tatC mutant cells on 2% SDS by TatB-mCherry and TatC-mCherry fusions. TatB, TatC, TatB-mCherry (TatB-mCh) and TatC-mCherry (TatC-mCh) were each expressed from pBAD33 vectors in TatABC wild-type, ΔtatC mutant (strain B1lK0), and ΔtatB mutant (strain BΦD) cells, as indicated. Cells grown in LB liquid media were adjusted to an OD600 of 0.1 and serially diluted and 5µl of each dilution was applied to agar plates containing 2% SDS and 0.1% arabinose. (TIF) [file pone.0069488.s003.tif]

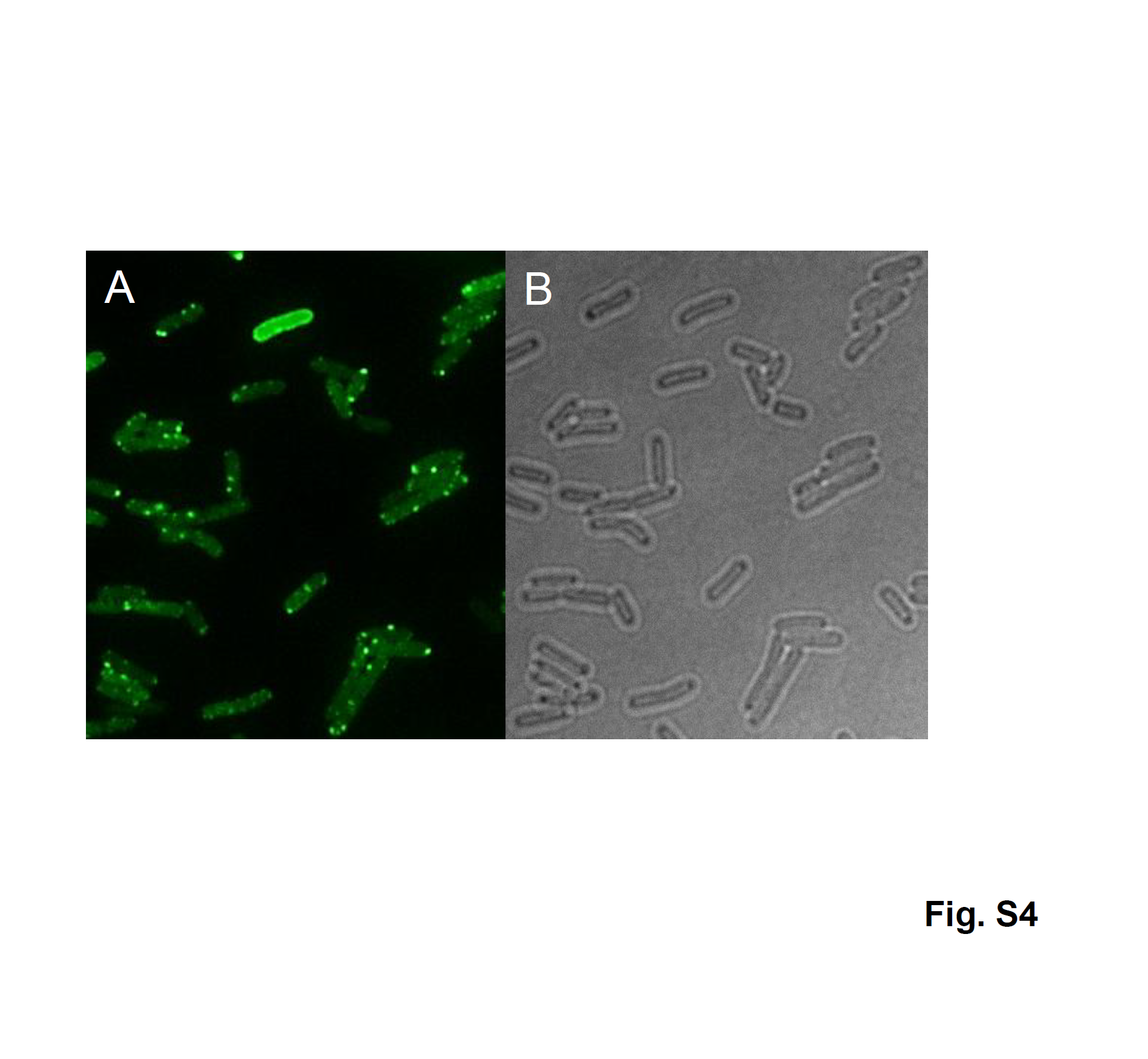

Supplement: Figure S4 — Localization of TatA-GFP in a TatABC wild-type strain co-expressing TorA-mCherry. (A) Same picture as shown in Figure 3C. (B) Phase contrast image of the same cells. (TIF) [file pone.0069488.s004.tif]
