# Supplementary material for: Substrate-Dependent Assembly of the Tat Translocase as Observed in Live Escherichia coli Cells
Source: PLoS One. 2013 Aug 2;8(8):e69488. doi: 10.1371/journal.pone.0069488 (PMC3732296; doi:10.1371/journal.pone.0069488)
Supplement: Table S1 — Primers used to construct the indicated plasmids. (DOCX) [file pone.0069488.s007.docx]

**Table S1. Primers used to construct the indicated plasmids**

| ***pPR1*** |  |
| --- | --- |
| 5´-SacI TatA Primer | GCGCGAGCTCATGGGTGGTATCAGTATTTGGC |
| 3´-BglII TatA Primer | GCGCAGATCTCACCTGCTCTTTATCGTGGC |
| 5-BlpI GfpMut1 | GCGCGTCGAGCATGAGTAAAGGAGAAGAACTTTTC |
| 3-XbaI GfpMut1 | GCGCGCTCTAGATTATTTGTATAGTTCATCCATGC |
| P-5-SufI-Linker | GATCTAAAATGTCCAGTCTTATCGGCCAGC |
| P-3-SufI-Linker | TCAGCTGGCCGATAAGACTGGACATTTTA |
| P-5´-SacI SD pBAD | CTTTAAGAAGGAGATGAGCT |
| P-3´-SacI SD pBAD | CATCTCCTTCTTAAAGGAGCT |
|  |  |
| ***pPR2*** |  |
| 5-BglII mCherry | CAGCAGATCTATGGTGAGCAAGGGCGAGGAG |
| 3`-XbaI C-mCherry | GCGCTCTAGATTACTTGTACAGCTCGTCCATGCCG |
| 5´-SacI TatB Primer | GCGCGAGCTCGTGTTTGATATCGGTTTTAGCG |
| 3´-BglII TatB Primer | GCGCAGATCTCGGTTTATCACTCGACGAAG |
|  |  |
| ***pPR3*** |  |
| 5´-SacI TatC Primer | GCGCGAGCTCATGTCTGTAGAAGATACTC |
| 3´-BglII TatC Primer | GCGCAGATCTTTCTTCAGTTTTTTCGCTTTC |
|  |  |
| ***pPR5*** |  |
| NcoI- TatA 5´-Primer | AACCATGGATGGGTGGTATCAGTATTTG |
| BamHI-TatA 3´-Primer | AAAGGATCCCACCTGCTCTTTATCGTGGC |
| P-5´-Linker | GATCTAAAATGTCCAGTCTTCGGATCCAGC |
| P-3´-Linker | TCAGCTGGATCCGAAGACTGGACATTTTA |
| NheI-cYfp 3´-Primer | AAAGCTAGCTTATTTGTATAGTTCATCCATGC |
| 5-BlpI GfpMut1 | GCGCGTCGAGCATGAGTAAAGGAGAAGAACTTTTC |
|  |  |
| ***pPR6*** |  |
| 5-P-XhoI SsrA-Tag | TCGAGGCAGCAAACGACGAAAACTACGCTTTAGCAGCTTAAC |
| 3-P-XhoI SsrA-Tag | TCGAGTTAAGCTGCTAAAGCGTAGTTTTCGTCGTTTGCTGCC |
|  |  |
| ***pPR7*** |  |
| 5-pASK TorA-mcherry BSAI | ATGGTAGGTCTCAAATGAACAATAACGATCTCTTTCAGGCAT |
| 3-pASK TorA-mcherry BSAI | ATGGTAGGTCTCAGCGCTCTTGTACAGCTCGTCCATGCCG |
|  |  |
| ***pPR8*** |  |
| 5-pASKIBA33 TorA-mCherry-SSrA | ATGGTAGGTCTCAAATGAACAATAACGATCTCTTTCAGGCAT |
| 3-pASKIBA33 TorA-mCherry-SSrA | ATGGTAGGTCTCAGCGCTTTAAGCTGCTAAAGCGTAGTTTTC |
